# Alcohol-Induced Acute Pancreatitis Complicated by Severe Direct Antiglobulin Test (DAT)-Negative Hemolytic Anemia and Thrombocytopenia: A Suspected Evans Syndrome-Like Immune Cytopenia

**DOI:** 10.7759/cureus.105011

**Published:** 2026-03-10

**Authors:** Roy Arslan Ahmed, Motaz Almahmood, Hatem Ahmed, Rita Ahmad, Shehab Fareed

**Affiliations:** 1 Internal Medicine, Tower Health Medical Group, Phoenixville, USA; 2 Internal Medicine, University of North Dakota, Fargo, USA; 3 Hematology and Medical Oncology, Hamad Medical Corporation, Doha, QAT

**Keywords:** acute pancreatitis, alcohol-related pancreatitis, dat-negative autoimmune hemolytic anemia, evans syndrome, hemolytic anemia, immune-mediated cytopenia, thrombocytopenia

## Abstract

We report a 28-year-old man with alcohol-induced interstitial acute pancreatitis (AP) who developed rapidly progressive anemia and thrombocytopenia beginning on hospital day two. Hemoglobin decreased from 16.5 g/dL on presentation to a nadir of approximately 5.2 g/dL, and the platelet count declined from 309×10^3^/µL to 12×10^3^/µL. Laboratory evaluation showed hemolysis with markedly elevated lactate dehydrogenase, undetectable haptoglobin, hyperbilirubinemia, and a peripheral smear demonstrating polychromasia with rare schistocytes and spherocytes, while the direct antiglobulin test (DAT) was negative on two occasions. Renal function, coagulation studies, and ADAMTS13 activity were normal, and broad infectious and autoimmune evaluation was unrevealing. Given the temporal association with pancreatitis, exclusion of thrombotic microangiopathy (TMA), disseminated intravascular coagulation, infection, and other evaluated causes, and hematologic improvement after intravenous immunoglobulin (IVIG) and corticosteroids, the working diagnosis was a suspected Evans syndrome-like immune cytopenia (immune thrombocytopenia with DAT-negative immune hemolysis) triggered by AP. Extended serologic testing for DAT-negative autoimmune hemolysis was negative, so the diagnosis remained a clinical working diagnosis. The patient was treated with transfusion support, IVIG, and intravenous methylprednisolone, with gradual hematologic recovery. This case underscores the importance of early hematologic evaluation and consideration of immunomodulatory therapy after exclusion of TMA and other common causes of hemolysis in patients with AP and severe cytopenias.

## Introduction

The pancreas secretes digestive enzymes that are normally activated in the duodenum; premature intrapancreatic activation can lead to autodigestion and acute pancreatitis (AP) [1,2]. AP has well-recognized etiologies, including gallstones, alcohol, hypertriglyceridemia, and medications [1,3]. In the United States, AP is a leading gastrointestinal cause of hospitalization [4]. Globally, alcohol has been estimated to account for approximately one-fifth of AP cases [5]. Thrombocytopenia and hemolytic anemia occurring together during AP warrant urgent evaluation for thrombotic microangiopathy (TMA), disseminated intravascular coagulation (DIC), hemolytic uremic syndrome (HUS), drug-induced cytopenias, and immune-mediated processes. We present a case of alcohol-induced interstitial AP complicated by profound hemolysis and severe thrombocytopenia without renal impairment, most consistent with a suspected Evans syndrome-like immune cytopenia temporally associated with AP.

## Case presentation

A 28-year-old man with chronic alcohol use disorder (approximately 12 beers daily, occasionally supplemented with two to three servings of whiskey) presented to the emergency department with sudden-onset epigastric abdominal pain after multiple episodes of non-bloody emesis. He denied melena, hematemesis, and recent diarrheal illness. He had no prior history of pancreatitis, liver disease, or hematologic disease and denied medication or supplement use, recent antibiotic exposure, and prior heparin exposure. He reported no known family history of hematologic or autoimmune disease. On arrival, vital signs were heart rate 68 beats/min, temperature 97.1°F (36.2°C), respiratory rate 18 breaths/min, blood pressure 161/90 mmHg, and oxygen saturation 94% on room air. Physical examination demonstrated epigastric and right upper quadrant tenderness without peritoneal signs. Murphy’s sign was negative. No jaundice, petechiae, or ecchymoses were noted. The patient was alert and oriented.

Initial laboratory testing showed markedly elevated lipase with transaminitis and leukocytosis, while creatinine, total bilirubin, hemoglobin, and platelet count were initially within reference range (Table 1). Serial hemoglobin, platelet count, and bilirubin trends were monitored during hospitalization. A viral hepatitis panel was negative. Based on the revised Atlanta classification, overall severity was most consistent with moderately severe AP (systemic complications without persistent organ failure).

**Table 1:** Selected quantitative laboratory findings with reference ranges AST, aspartate aminotransferase; ALT, alanine aminotransferase; LDH, lactate dehydrogenase; ARC, absolute reticulocyte count; ADAMTS13, a disintegrin and metalloproteinase with thrombospondin type 1 motif, member 13

Laboratory test	Result	Reference range
Lipase	>3,500 U/L	13-60 U/L
AST	191 U/L	10-40 U/L
ALT	272 U/L	7-56 U/L
White blood cell count	18,000/uL	4,000-11,000/uL
Hemoglobin	16.5 g/dL	13.5-17.5 g/dL
Platelet count	309x10^3^/uL	150-400x10^3^/uL
Creatinine	0.89 mg/dL	0.74-1.35 mg/dL
Total bilirubin	0.5 mg/dL	0.2-1.2 mg/dL
Ferritin	1,564 ng/mL	30-400 ng/mL
Blood alcohol level	75 mg/dL	Negative
LDH	2,536 U/L	140-280 U/L
Haptoglobin	<10 mg/dL	30-200 mg/dL
Reticulocytes	5.5% (ARC 133x10^9^/L)	0.5%-2.5% (ARC 25-100 x10^9^/L)
Peak bilirubin	10 mg/dL (direct 6.2 mg/dL)	Total 0.2-1.2 mg/dL; direct 0.0-0.3 mg/dL
ADAMTS13 activity	≥70%	≥67%

The patient was treated with aggressive intravenous fluids and supportive care. The first routine inpatient laboratory draw (hospital day one) remained similar to the presentation values. On hospital day two, he developed rapidly progressive thrombocytopenia and worsening anemia. A single prophylactic dose of enoxaparin was administered for venous thromboembolism prophylaxis and then discontinued when thrombocytopenia was recognized; venous thromboembolism prophylaxis thereafter consisted of sequential compression devices only. The patient received aggressive intravenous fluids during the first 48 hours of hospitalization, and the early decline in hemoglobin may have been partially dilutional. However, the subsequent rapid decline was accompanied by laboratory and smear evidence of hemolysis (elevated lactate dehydrogenase, undetectable haptoglobin, rising bilirubin, reticulocytosis, and hemolytic features on peripheral smear), supporting true hemolysis rather than dilution alone. There was no clinical evidence of overt bleeding during hospitalization (no hematemesis, melena, or hemodynamic instability), and fecal occult blood testing was negative.

**Figure 1:**
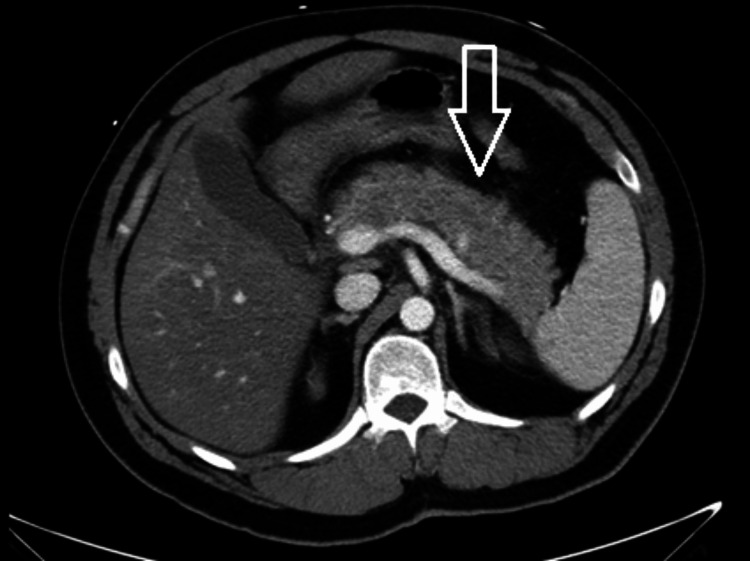
Contrast-enhanced CT of the abdomen and pelvis on hospital day one Representative axial CT image showing acute interstitial pancreatitis with peripancreatic inflammatory change on initial imaging. The arrow indicates an area of peripancreatic inflammatory change.

**Figure 2:**
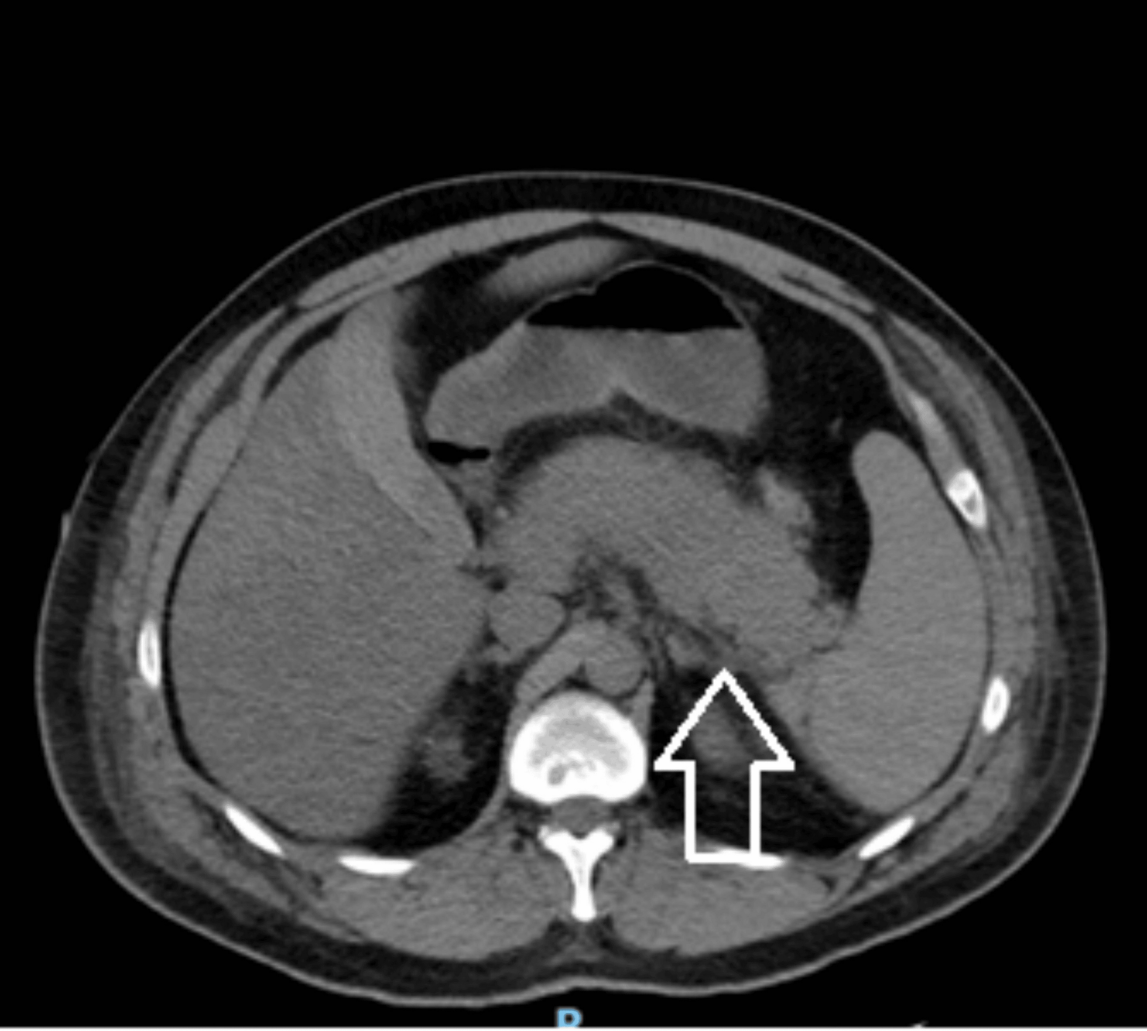
Follow-up contrast-enhanced CT of the abdomen and pelvis on hospital day four Representative axial CT image from serial imaging demonstrating evolving pancreatitis-related inflammatory changes during hospitalization. The arrow indicates an area of peripancreatic inflammatory change.

**Figure 3:**
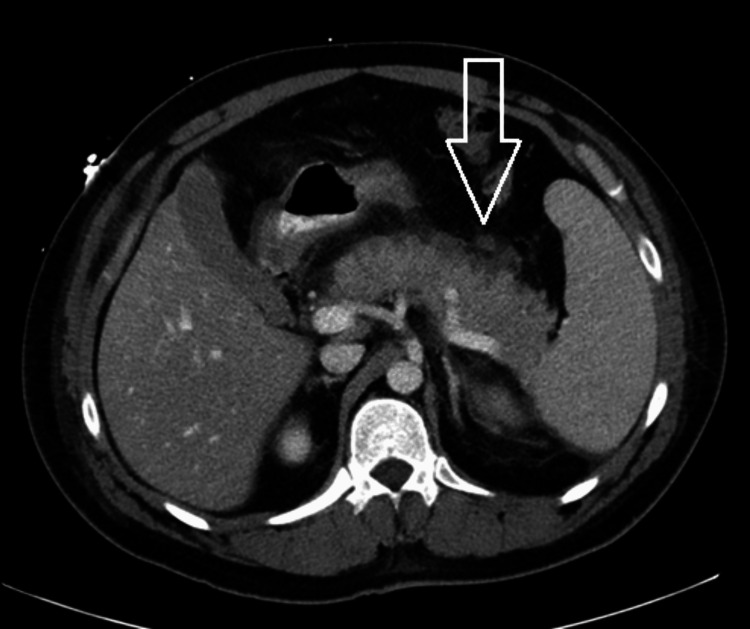
Follow-up contrast-enhanced CT of the abdomen and pelvis on hospital day eight Representative axial CT image from later follow-up showing persistent but improving pancreatitis-related inflammatory changes on serial imaging. The arrow indicates an area of peripancreatic inflammatory change.

**Figure 4:**
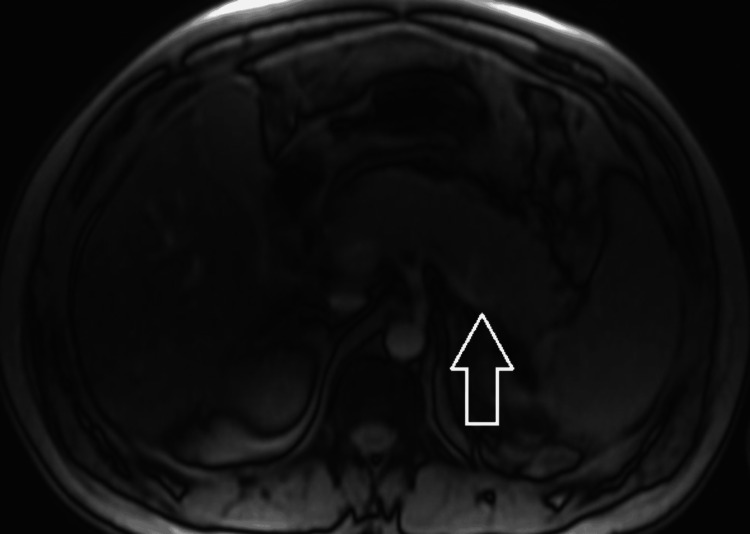
MRI/MRCP of the abdomen during early hospitalization Representative axial MRI/MRCP image obtained during early hospitalization. The arrow indicates an area of peripancreatic inflammatory change. MRCP, magnetic resonance cholangiopancreatography

CT of the abdomen and pelvis demonstrated acute interstitial pancreatitis without cholelithiasis, biliary ductal dilation, or pancreatic necrosis and was repeated on hospital days four and eight. Right upper quadrant ultrasound (hospital day two) demonstrated mild gallbladder wall thickening with sludge, hepatomegaly with diffuse fatty infiltration, mild ascites, and no biliary dilation. MRI/magnetic resonance cholangiopancreatography (MRCP) of the abdomen (hospital day three) confirmed AP with hepatic steatosis, without biliary obstruction or pancreatic necrosis. Representative imaging studies are shown in Figures 1-4.

**Figure 5:**
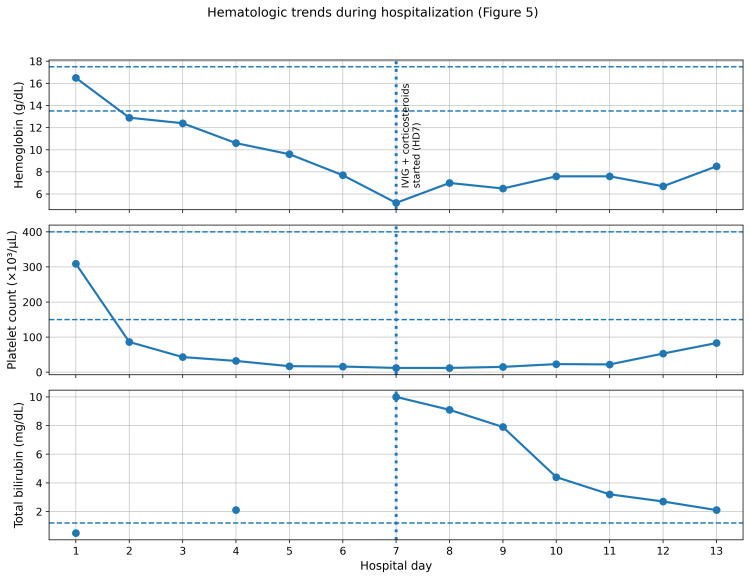
Serial hemoglobin, platelet count, and total bilirubin values are shown by hospital day Dashed horizontal lines indicate the reference ranges for hemoglobin (13.5-17.5 g/dL), platelet count (150-400×10³/µL), and total bilirubin (upper limit 1.2 mg/dL). The vertical dotted line indicates initiation of IVIG and corticosteroid therapy on hospital day seven. IVIG, intravenous immunoglobulin

Coagulation studies (prothrombin time (PT)/international normalized ratio (INR) and activated partial thromboplastin time (aPTT)) remained within normal limits. Fibrinogen was mildly elevated at 505 mg/dL (reference range 213-486 mg/dL), and D-dimer was elevated. Peripheral blood smear demonstrated polychromasia with rare schistocytes and spherocytes, anisocytosis, and macrocytosis, with only limited microangiopathic features. The direct antiglobulin test (DAT; polyspecific antihuman globulin (AHG)) was negative on two occasions; monospecific testing and an enhanced evaluation for DAT-negative autoimmune hemolysis were also negative. Heparin-induced thrombocytopenia (HIT) testing (immunoassay and serotonin release assay) was negative. Selected quantitative laboratory findings with reference ranges are summarized in Table 1, and serial hemoglobin, platelet count, and bilirubin trends are shown in Figure 5.

A broad evaluation for infectious, autoimmune, and malignant etiologies was unrevealing, including negative cytomegalovirus (CMV), Epstein-Barr virus (EBV), human immunodeficiency virus (HIV), viral hepatitis, and mycoplasma testing; normal copper and vitamin B12/folate levels; normal triglyceride level; negative urine toxicology; and negative blood cultures. A disintegrin and metalloproteinase with thrombospondin type 1 motif, member 13 (ADAMTS13) activity was normal.

Given progressive anemia and thrombocytopenia with laboratory and smear evidence of hemolysis, hematology was consulted as the differential diagnosis was narrowed through serial smear review and exclusion of major mimics (including TMA, DIC, HIT, and evaluated infectious causes). The working diagnosis was a suspected Evans syndrome-like immune cytopenia (immune thrombocytopenia with DAT-negative immune hemolysis) temporally associated with AP. Packed red blood cell transfusions (a total of 4 units) were administered for severe anemia. Intravenous immunoglobulin (IVIG) and intravenous methylprednisolone were initiated on hospital day seven during hematologic nadir (Figure 5). The intravenous methylprednisolone regimen consisted of 1 mg/kg/day for three days followed by 0.5 mg/kg/day for three days. After early hematologic improvement (with recovery in platelet count and stabilization/improvement in hemoglobin and hemolysis markers), the patient was transitioned at discharge (hospital day 13) to oral prednisone 40 mg daily with a planned taper over four weeks under hematology guidance, with dose adjustments based on serial complete blood count and hemolysis laboratory monitoring. Hematologic recovery began after initiation of immunomodulatory therapy, although causality cannot be proven in a single case, and concurrent transfusion support may have influenced immediate post-treatment values.

The patient remained hemodynamically stable and afebrile throughout hospitalization. Following initiation of IVIG and corticosteroids on hospital day seven, platelet count and hemoglobin began to recover over the subsequent days, with downtrending bilirubin and hemolysis markers (Figure 5). He was discharged on an oral prednisone taper with counseling on alcohol cessation and outpatient hematology and gastroenterology follow-up. At a two-week follow-up, hemoglobin and platelet count had normalized, and hemolysis markers had returned to near baseline.

## Discussion

This case describes alcohol-induced interstitial AP complicated by profound anemia and severe thrombocytopenia beginning on hospital day two. The combination of hemolysis and thrombocytopenia in the setting of pancreatitis immediately raises concern for TMA on the thrombotic thrombocytopenic purpura (TTP)/HUS spectrum, as pancreatitis has been reported to precipitate TMA via systemic inflammation and endothelial injury [6]. However, several features in this patient argued against classic TMA: only rare schistocytes were observed, renal function remained normal, and coagulation studies were normal with a mildly elevated fibrinogen level, making DIC unlikely. Although early hemoglobin decline in AP may be influenced by hemodilution during fluid resuscitation, there was no clinical evidence of overt bleeding, and fecal occult blood testing was negative; the later accelerated decline occurred with robust biochemical and smear evidence of hemolysis, making blood loss or hemodilution alone unlikely.

Autoimmune hemolytic anemia (AIHA) and immune thrombocytopenia can occur concurrently as Evans syndrome. Importantly, approximately 5%-10% of AIHA cases may have a negative DAT depending on assay sensitivity and antibody class [7]. Because IgA or low-affinity antibodies can be missed by standard DAT methods, extended testing (when available) may help characterize immune hemolysis in DAT-negative cases [7,8]. In this patient, the peripheral smear suggested hemolysis with spherocytes, hemolysis markers were markedly abnormal, and platelet recovery followed IVIG and corticosteroids, supporting a suspected Evans syndrome-like immune cytopenia temporally associated with pancreatitis. Published reports linking AP to immune cytopenias are rare. We identified a prior report of pancreatitis-associated Fisher-Evans syndrome; however, we did not identify clear prior case reports specifically describing DAT-negative AIHA temporally associated with AP [9]. In this case, repeat polyspecific DAT testing and subsequent monospecific anti-IgG/anti-C3d testing were negative, and extended serologic testing for DAT-negative autoimmune hemolysis was also negative. Accordingly, the diagnosis remained a clinical working diagnosis based on hemolysis markers and smear findings, exclusion of alternative etiologies, and hematologic improvement after IVIG and corticosteroids.

HIT was considered, given the temporal relationship to a single enoxaparin dose; however, rapid-onset HIT generally requires heparin exposure within the preceding 100 days, and isolated HIT would not explain the simultaneous severe hemolysis. Drug-induced cytopenias were also considered, but were less likely given limited medication exposure. Other causes of hemolysis and thrombocytopenia, including HUS, viral-associated hemophagocytic syndromes, and occult infection, were evaluated and not supported by available testing.

Management in this case was guided by clinical evolution and exclusion of high-risk mimics. Supportive care for pancreatitis was continued, with transfusion for severe anemia. Immunomodulatory therapy with IVIG and corticosteroids was chosen, considering suspected immune thrombocytopenia with immune hemolysis. Empiric antibiotics in pancreatitis remain controversial; routine prophylaxis is generally not recommended in the absence of suspected or proven infection (including infected necrosis), although antibiotics may be considered when there is clinical concern for infection or sepsis [3,10]. In this case, no antibiotics were administered because there was no clinical, microbiologic, or imaging evidence of infection.

The pathophysiologic link between AP and concurrent immune-mediated cytopenias remains incompletely defined [11]. AP is associated with marked systemic inflammation and cytokine dysregulation, which may contribute to hematologic complications; however, a direct mechanism for pancreatitis-triggered DAT-negative immune hemolysis with thrombocytopenia has not been established [11,12]. In this context, early hematology consultation and timely consideration of immunomodulatory therapy may be reasonable after exclusion of TMA, DIC, infection, and other common causes of hemolysis and thrombocytopenia.

## Conclusions

Severe hemolytic anemia with profound thrombocytopenia can complicate alcohol-induced AP even without pancreatic necrosis or renal failure. When common causes such as TMA/TTP-HUS, DIC, infection, and drug-induced cytopenias are excluded, a suspected immune-mediated cytopenia, including a possible Evans syndrome-like process with direct antiglobulin test (DAT)-negative immune hemolysis, should be considered. In this case, the diagnosis remained a clinical working diagnosis despite negative repeat polyspecific and monospecific DAT testing and negative extended serologic evaluation for DAT-negative autoimmune hemolysis. Early immunomodulatory therapy with IVIG and corticosteroids, alongside supportive care, may lead to hematologic recovery.

## References

[1] Forsmark CE, Vege SS, Wilcox CM (2016). Acute pancreatitis. N Engl J Med.

[2] Banks PA, Freeman ML (2006). Practice guidelines in acute pancreatitis. Am J Gastroenterol.

[3] Tenner S, Baillie J, DeWitt J, Vege SS (2013). American College of Gastroenterology guideline: management of acute pancreatitis. Am J Gastroenterol.

[4] Peery AF, Crockett SD, Murphy CC (2022). Burden and cost of gastrointestinal, liver, and pancreatic diseases in the United States: update 2021. Gastroenterology.

[5] Zilio MB, Eyff TF, Azeredo-Da-Silva AL, Bersch VP, Osvaldt AB (2019). A systematic review and meta-analysis of the aetiology of acute pancreatitis. HPB (Oxford).

[6] Swisher KK, Doan JT, Vesely SK (2007). Pancreatitis preceding acute episodes of thrombotic thrombocytopenic purpura-hemolytic uremic syndrome: report of five patients with a systematic review of published reports. Haematologica.

[7] Kamesaki T (2022). Diagnostic algorithm for classification and characterization of direct antiglobulin test-negative autoimmune hemolytic anemia with 1-year clinical follow-up. Transfusion.

[8] Gergal Gopalkrishna Rao SR (2024). A case of Coombs-negative primary warm autoimmune hemolytic anemia due to IgA antibody that responded well to rituximab and steroids. Cureus.

[9] Domínguez-Pérez R, Arrazola-García FV (2021). Fisher-Evans syndrome associated to acute pancreatitis. Med Crit (Col Mex Med Crit).

[10] Baron TH, DiMaio CJ, Wang AY, Morgan KA (2020). American Gastroenterological Association clinical practice update: management of pancreatic necrosis. Gastroenterology.

[11] Venkatesh K, Glenn H, Delaney A, Andersen CR, Sasson SC (2022). Fire in the belly: a scoping review of the immunopathological mechanisms of acute pancreatitis. Front Immunol.

[12] Rodberg K (2022). DAT-negative autoimmune hemolytic anemia. Hematol Oncol Clin North Am.

